# Disease progression modeling of Alzheimer’s disease based on variational probability principal component analysis

**DOI:** 10.1371/journal.pone.0342549

**Published:** 2026-03-30

**Authors:** Xin Xiong, Ximin Wang, Chenyang Zhu, Jianfeng He, for the Alzheimer’s Disease Neuroimaging Initiative

**Affiliations:** Faculty of Information Engineering and Automation, Kunming University of Science and Technology, Kunming, China; University of Pennsylvania Perelman School of Medicine, UNITED STATES OF AMERICA

## Abstract

Alzheimer’s disease (AD) is a neurodegenerative disorder and the leading cause of dementia. Early diagnosis and monitoring of disease progression are crucial for effective intervention. This study presents a novel disease progression model based on Variational Probabilistic Principal Component Analysis (VPPCA), which uses a Bayesian framework for dimensionality reduction and uncertainty quantification. By analyzing 1,021 amyloid-positive patients from the Alzheimer’s Disease Neuroimaging Initiative (ADNI) database, we extracted 25 features, including CSF (ABETA, TAU, PTAU), PET (FDG, AV45), and MRI volumetrics, along with cognitive and functional assessments. VPPCA compresses these multi-modal biomarkers into a single first principal component score (VPPCA1), which serves as a measure of disease progression. To ensure biological grounding and avoid circularity, we demonstrated that a VPPCA1 model using only non-cognitive features (CSF, PET, MRI, demographics) correlates strongly with cognitive decline (r = 0.658 with ADAS-Cog13), confirming that it captures genuine pathological progression rather than simply reflecting cognitive assessments. Block-wise feature ablation revealed that multi-modal integration is essential, with cognitive features showing the highest importance (0.1064), though all modalities contribute complementarily. In classification tasks, VPPCA exhibited strong performance with ROC-AUC values of 0.990 (CN vs Dementia), 0.774 (CN vs MCI), and 0.785 (MCI vs Dementia). A Bayesian hierarchical longitudinal model effectively captured patient-specific progression trajectories, offering personalized predictions of future disease states. VPPCA outperforms Probabilistic PCA (PPCA) by providing uncertainty quantification, with patient-specific confidence levels (σ = 0.086–0.136), which correlate with data quality, enabling automatic risk stratification. This work demonstrates that VPPCA offers a robust, biologically-grounded framework for modeling AD progression, providing actionable uncertainty quantification that improves clinical decision support and facilitates personalized care.

## Introduction

Alzheimer’s disease (AD) is a neurodegenerative disease and the leading cause of dementia. The number of patients is expected to exceed 7 million by 2030 [1]; by 2050, the global prevalence will triple and is expected to affect more than 100 million people [2]. Early diagnosis of patients can be achieved by understanding the underlying pathological mechanisms, identifying multiple pathogenic and protective genes, and introducing lifestyle changes for intervention using new blood analysis methods. Novel biomarkers obtained by PET scans and plasma analysis of β-amyloid and phosphorylated tau protein have great potential in AD research and clinical diagnosis. Although lifestyle may not directly affect the pathological mechanisms of AD, it may still have a positive impact on patients. It is important to distinguish between biological biomarkers (such as amyloid and tau proteins measured via CSF or PET imaging, and brain structural changes from MRI) and clinical outcome assessments (such as cognitive test scores and functional rating scales), as they capture different aspects of disease progression.

The potential status of AD patients can be described from the biological perspective based on changes in relevant imaging biomarkers. Clinicians can use a trimodal staging system to diagnose AD dementia and determine the intermediate state between individuals with normal cognition (CN) and mild cognitive impairment (MCI) [3]. Kumar et al. [4] have explored predicting correct diagnosis from biomarkers, and Fisher et al. [5] have explored describing individual conditions from a single cognitive test, but these methods may be limited by the different sensitivities of the tests. Jack et al. [6] proposed a dynamic hypothesis model that combines disease stage with individual AD biomarkers, and judges the severity of clinical symptoms by observing whether the biomarkers are abnormal. Martin et al. [7] used probabilistic principal component analysis (PPCA) to describe the potential state of individuals in the Alzheimer’s disease continuum as a single score for a set of related biomarkers. This score can directly reflect the state of AD disease progression and can also be interpreted longitudinally based on time-shifted and multiple visits. The method they proposed can quickly discover low-dimensional representations of variability in biomarkers, and this unsupervised method is comparable to supervised models that require longitudinal data and has strong correlation with other more complex disease progression models.

However, this method still has some limitations. First, the method only selects quantitative biomarkers, and in fact other demographic information is equally important, such as age, years of education, number of APOE ε4 allele carriers, and dementia cognitive test scales. Secondly, regarding the interpretability of this method, although the first principal component was shown to be diagnostic in the latent space in 2021 [8], the authors did not explain whether the use of only the first principal component masks other variations in the biomarker. For PPCA [9], they only used this method to handle missing data and solve the noise of the score, but the uncertainty of the first principal component score was not handled.

Therefore, we proposed variational probability principal component analysis (VPPCA), which can not only estimate missing data, but also construct scores in the latent space from various biomarkers related to Alzheimer’s disease. This value can be summarized as a progression score for the longitudinal model of disease progression, highlighting the changes in biomarkers on the AD continuum to reflect the disease progression of Alzheimer’s patients. This method can also be used for the three-mode staging system classification of the AD continuum. In response to the uncertainty of the score, this method uses variational Bayesian inference to approximate the posterior distribution of the noise of the score obtained in the latent dimension, which is the prior distribution of the noise of the single trajectory score in the longitudinal hierarchical disease progression model.

## Materials and methods

### Alzheimer’s disease neuroimaging initiative (ADNI)

The data used in this article are from the Alzheimer’s Disease Neuroimaging Initiative (ADNI) database. Our study collected 16,422 records from 2,431 patients, covering data from four stages: ADNI-1, ADNI-2, ADNI-GO, and the latest ADNI-3. On this basis, 1,021 patients who were amyloid-positive at baseline were selected as research subjects, representing individuals in the AD continuum [10]. We selected a series of validated biomarkers for analysis, including amyloid, microtubule-associated protein tau, a series of cognitive test results, and specific brain area volume markers. Table 1 shows the basic demographic information of the patients and their related biomarker data statistics at baseline.

**Table 1:** Baseline demographic information of patients who were amyloid-positive at baseline diagnosis.

	CN	MCI	Dementia
**N**	260	510	251
**Sex (Female)**	146	206	102
**Age**	73.5 ± 6.2	73.0 ± 7.2	74.0 ± 8.3
**APOE ε4(0)**	144(55.4%)	189(37.1%)	68(27.1%)
**APOE ε4(1)**	96(36.9%)	231(45.3%)	125(49.8%)
**APOE ε4(2)**	17(6.5%)	80(15.7%)	56(22.3%)
**APOE ε4(unknown)**	3(1.2%)	10(2.0%)	2(0.8%)
**Years of education**	16.5 ± 2.4	16.1 ± 2.8	15.5 ± 2.9
**Ventricles**	36877 ± 20207	42183 ± 23270	49931 ± 24148
**Hippocampus**	7448 ± 880	6718 ± 1074	5864 ± 1038
**Whole Brain**	1039889 ± 110070	1036920 ± 109180	994893 ± 117573
**Entorhinal**	3928 ± 625	3515 ± 742	2890 ± 749
**Fusiform**	18321 ± 2301	17685 ± 2740	15771 ± 2762
**Mid Temp**	20578 ± 2696	19630 ± 2901	17455 ± 3197
**FDG**	1.3 ± 0.1	1.2 ± 0.1	1.0 ± 0.1
**FAQ**	0.2 ± 0.7	3.6 ± 4.1	13.0 ± 6.7
**AV45**	1.2 ± 0.2	1.3 ± 0.2	1.4 ± 0.2
**ABETA**	802 ± 270	722 ± 237	605 ± 212
**TAU**	246 ± 105	311 ± 138	370 ± 142
**PTAU**	23.8 ± 11.2	31.0 ± 15.6	37.3 ± 15.6
**CDRSB**	0.04 ± 0.1	1.6 ± 1.0	4.4 ± 1.6
**ADAS13**	9.0 ± 4.4	17.4 ± 6.8	30.3 ± 8.2
**ADASQ4**	2.8 ± 1.8	5.8 ± 2.5	8.7 ± 1.5
**RAVLT immediate**	44.8 ± 10.0	33.0 ± 9.8	22.9 ± 7.3
**RAVLT learning**	6.0 ± 2.5	4.0 ± 2.6	1.9 ± 1.8
**RAVLT forgetting**	4.0 ± 2.8	4.9 ± 2.3	4.5 ± 1.8
**RAVLT perc. Forgetting**	36.9 ± 27.6	64.2 ± 31.2	88.4 ± 22.5
**LDELTOTAL**	13.0 ± 3.1	5.5 ± 3.5	1.4 ± 1.9
**DIGITSCOR**	46.0 ± 9.2	35.6 ± 10.8	26.9 ± 12.3
**TRABSCOR**	87.7 ± 44.3	122.7 ± 68.0	198.4 ± 87.0

### Data preprocessing

All experiments in this paper focused on patients who were amyloid-positive, who were considered to be on the AD continuum. All our data were obtained using the ADNIMERGE R package, and only patients who were amyloid-positive were selected for the primary analysis. Amyloid positivity was determined based on each patient’s baseline cerebrospinal fluid (CSF) amyloid-β (1–42) concentration. Two validated immunoassay platforms were used in ADNI, each with established positivity thresholds: Roche Elecsys immunoassay: Aβ42 < 1100 pg/mL indicates amyloid positivity [11]; INNO-BIA AlzBio3 immunoassay: Aβ42 < 192 pg/mL indicates amyloid positivity [12].

Lower CSF Aβ42 concentrations reflect greater sequestration of amyloid-β into cerebral plaques, resulting in reduced levels in the CSF. This inverse relationship between CSF Aβ42 and brain amyloid burden is consistent with the amyloid cascade hypothesis of AD and has been validated against amyloid PET imaging.

Patients were classified as amyloid-positive if their Aβ42 concentration fell below the platform-specific threshold on either assay. Of the initial 2,431 patients, 1,021 (42.0%) were identified as amyloid-positive at baseline using these criteria.

CSF biomarker data were obtained from the ADNIMERGE R package. Specifically, INNO-BIA AlzBio3 measurements were retrieved from the UPENNBIOMK_MASTER file, and Roche Elecsys measurements were extracted from the UPENNBIOMK9, UPENNBIOMK10, and UPENNBIOMK12 files.

For feature selection, we chose measures commonly used in disease progression modeling that are available for most ADNI patients. Our feature set comprises:

(1) Biological biomarkers: CSF biomarkers: β-amyloid protein (ABETA), microtubule-associated protein tau (TAU), and phosphorylated tau (PTAU), which are established markers of neurodegeneration [13]; PET imaging: Fluorodeoxyglucose (FDG) uptake, quantified using the Florbetapir compound for β-amyloid deposition. While Pittsburgh Compound-B and Florbetaben also measure β-amyloid, we excluded them due to small sample sizes and minimal differences from Florbetapir [14]; MRI volumetrics: Volumes of ventricles, hippocampus, entorhinal cortex, whole brain, fusiform gyrus, and medial temporal gyrus [15].
(2) Clinical outcome assessments:
(3) Cognitive measures: Clinical Dementia Rating Scale Sum of Boxes (CDR-SB), Alzheimer’s Disease Assessment Scale cognitive subscale (ADAS-Cog13), ADAS-Cog delayed recall (ADASQ4), Rey Auditory Verbal Learning Test immediate recall (RAVLT Immediate), RAVLT Learning score, RAVLT Forgetting score, RAVLT Percent Forgetting, Logical Memory Delayed Recall (LDELTOTAL), Digit Symbol Substitution Test (DIGITSCOR), and Trail Making Test (TRABSCOR) [16]; Functional assessment: Functional Assessment Questionnaire (FAQ).
(4) Demographic and genetic information: Age (AGE), years of education (PTEDUCAT), and APOE ε4 allele status.

These tests assess the clinical manifestations of AD across cognitive, functional, and demographic domains. To maintain clarity, we hereafter refer to the complete set of 25 measures as ‘features’, distinguishing biological biomarkers from clinical assessments where necessary.

The final feature set contained 25 variables (missing rate<40%), covering biomarkers, clinical assessments, and demographic information (detailed missing rate statistics are shown in Supplementary S1 Table). Among the 1021 amyloid-positive patients at baseline, most variables had low missing rates: cognitive assessment data were nearly complete (missing rate 0–3.53%), MRI volume measurements had low missing rates (3.82–15.18%), and cerebrospinal fluid biomarkers had moderate missing rates (18.51%). The highest missing rates were for AV45-PET (39.76%) and DIGITSCOR (38.89%), both below our pre-set 40% threshold.

To reduce the expected heterogeneity of brain region volumes due to age and intracranial volume, we adjusted the volumes of six brain regions obtained from MRI scans according to age and intracranial volume (ICV) at baseline [17]. This approach allowed us to focus on the common trends in biomarker evolution across affected individuals. All biomarkers were scaled using z-score normalization to reduce differences in scale and magnitude.

Finally, amyloid-positive patients were randomly divided into two groups, training and test sets, of equal size. This grouping was first used for normalization purposes, and the test set was renormalized according to the covariance matrix of the biomarkers processed using VPPCA.

### Variational probabilistic principal component analysis (VPPCA)

In the previous section, we extracted 25 features related to Alzheimer’s disease from the original data. In order to capture the differences in disease states in the dataset, we used VPPCA [18] to project the original data into a low-dimensional latent space, which can retain the maximum variability in the data. VPPCA provides a more flexible and robust way to reduce the dimension and extract features from data by introducing a probabilistic model and a variational inference framework. The estimation of model parameters is no longer a single point estimate, but exists in the form of probability distribution, which increases the uncertainty representation and interpretability of the model.

#### Variational inference for Alzheimer’s disease biomarker analysis.

In the VPPCA model, we use variational inference to estimate the potential changes in biomarkers associated with Alzheimer’s disease. Considering the complexity of biomarker data in Alzheimer’s disease research, including the high dimensionality and missing value problems of the data, variational inference provides us with a method to efficiently estimate model parameters.

In order to maximize the log-likelihood function of the observed data X, where X contains the observed values of 25 features related to the progression of Alzheimer’s disease, a set of latent variables Z (the position of each patient on the AD progression continuum, which reflects the patient’s cognitive state and the degree of disease progression) and model parameters θ (biomarker loading, which can represent the weight of the influence of different biomarkers on the patient’s potential disease progression state) are introduced, and a simplified variational distribution q(Z,θ) is introduced to approximate the true posterior distribution p(Z,θ|X).

The goal of variational inference is to find the optimal variational distribution $q^{*}(Z,\theta )$ so that it is as close as possible to the true posterior distribution p(Z,θ|X). First, the variational method introduces a set of approximate densities Q(Z,θ), which is a set of probability densities of latent variables. The goal of variational inference is to find the variational distribution q(Z,θ) that minimizes the Kullback-Leibler (KL) divergence between q(Z,θ) and the true posterior p(𝑍, 𝜃|𝑋), thereby making q as close as possible to the true posterior. The KL divergence is a measure of dissimilarity between two probability distributions. Formula (1) formally states this optimization objective:


$$q^{*}(Z,\theta )=\begin{array}{c} argmin\\ q(Z,\theta )\in Q \end{array}KL\left(q(Z,\theta )\|p\left(Z,\theta |X\right)\right)$$ (1)

The KL divergence formula is


$$KL\left(q(Z,\theta )\|p(Z,\theta |X)\;\right)=\int q(Z,\theta )\text{ln}\frac{q(Z,\theta )}{p(Z,\theta |X)}d\theta$$ (2)

Using Bayes’ theorem and observed data X={A,B}, KL divergence can be rewritten as


$$KL\left(q(Z,\theta )\|p(Z,\theta |X)\;\right)=\int q(Z,\theta )\left[\text{ln}q(Z,\theta )-\text{ln}\frac{p\left(Z,\theta |X\;\right)p(X)}{p(X)}d\theta \right]$$ (3)

L(Q)=∫q(Z,θ)[−lnq(Z,θ)+lnp(Y|(Z,θ),A)+lnp(Z,θ)]dθ (4)

This formulation (equation 4) decomposes the ELBO into three interpretable terms: the expected log-likelihood of the observed data, the prior log-probability of the latent variables and parameters, and the negative entropy of the variational distribution. Maximizing the ELBO with respect to q is equivalent to minimizing the KL divergence between q and the true posterior, as shown in equation (5).

ELBO (Evidence Lower Bound) is


lnp(D)∫q(Z,θ)dθ=KL(q(Z,θ)‖p(Z,θ|D ) )+ L(Q) (5)

In equation (3), (*X*) is constant with respect to q, and the KL divergence 𝐾𝐿(q(𝑍, 𝜃)‖p(𝑍, 𝜃|𝑋)) ≥ 0 by Gibbs’ inequality. From equation (5), we observe that: ln (𝑋) = 𝐾𝐿(q(𝑍, 𝜃)‖p(𝑍, 𝜃|𝑋)) + 𝐿(Q).

Since ln (*X*) is fixed and the KL divergence is non-negative, maximizing 𝐿(Q) (the Evidence Lower Bound, ELBO) is equivalent to minimizing the KL divergence 𝐾𝐿(q(𝑍, 𝜃)‖p(𝑍, 𝜃|𝑋)). Therefore, variational inference seeks to find the optimal variational distribution q*(𝑍, 𝜃) by maximizing the ELBO.

In order to find the most suitable variational cluster Q(θ) and the optimal parameters to minimize L(Q), we introduce the mean field variational family decomposition $q(\theta )=\prod_{i}q_{i}(\theta_{i})$, where the latent variables Z are independent of each other and each variable is controlled by a different factor in the variational density. By choosing Gaussian distribution as the distribution of $q_{i}(\theta_{i})$, L(Q) can be minimized on $q_{i}(\theta_{i})$, and the variational distribution in the form of Gaussian distribution is


$$q_{i}\left(\theta_{i}\right)=\frac{\text{exp}\{E_{\frac{q(\theta )}{q\left(\theta_{i}\right)}}[\text{In}p(X,\theta )]\}}{\int \text{exp}\{E_{\frac{q(\theta )}{q\left(\theta_{i}\right)}}[\text{In}p(X,\theta )]\}d\theta_{i}}$$ (6)

where $E_{\frac{q(\theta )}{q\left(\theta_{i}\right)}}$ represents the expectation over all parameters except $\theta_{i}$, p(X,θ) is the joint probability of the model under the observed data X(biomarker) and parameters θ(biomarker loading),


$$\frac{q(\theta )}{q\left(\theta_{i}\right)}=\prod_{k\neq i}q_{k}(\theta_{k})$$ (7)

Specify the prior distribution on $\text{x},\alpha ,\mu ,\tau =\sigma^{-\text{2}}$ as


p(θ)=p(X)p(W) p(x) p(α)p(μ)p(τ) (8)

The update formula of the fixed-point iteration algorithm is set as


$$\overset{\left(n\right)}{\underset{\text{x}}{m}}=\left\langle \tau \right\rangle \Sigma_{\text{x}}\left\langle {\text{W}}^{T}\right\rangle \left(t_{n}-\left\langle \mu \right\rangle \right)$$ (9)

$$\Sigma_{\text{x}}={\left(\text{I}+\left\langle \tau \right\rangle \left\langle {\text{W}}^{T}\text{W}\right\rangle \right)}^{-\text{1}}$$ (10)

$$m_{\mu }=\left\langle \tau \right\rangle \Sigma_{\mu }\sum_{n=\text{1}}^{N}\left(t_{n}-\left\langle W\right\rangle \left\langle {\text{x}}_{n}\right\rangle \right)$$ (11)

$$\Sigma_{\mu }={\left(\beta +N\left\langle \tau \right\rangle \right)}^{-\text{1}}\text{I}$$ (12)

$$\overset{\left(k\right)}{\underset{\text{w}}{m}}=\left\langle \tau \right\rangle \Sigma_{\text{w}}\sum_{n=\text{1}}^{N}\left\langle {\text{x}}_{n}\right\rangle \left(t_{nk}-\left\langle \mu_{k}\right\rangle \right)$$ (13)

$$\Sigma_{\text{w}}={\left(\text{diag}\left\langle \alpha \right\rangle +\left\langle \tau \right\rangle \sum_{n=\text{1}}^{N}\left\langle {\text{x}}_{n}\overset{T}{\underset{n}{\text{x}}}\right\rangle \right)}^{-\text{1}}$$ (14)

Use the fixed-point iteration algorithm to find the optimal parameters x,W,α,μ,$\sigma^{\text{2}}$. After setting the initial values, gradually adjust the variables until convergence.

#### Estimating missing values.

Missing values are common in longitudinal clinical datasets like ADNI [19]. Both biological biomarkers (e.g., CSF measures requiring lumbar puncture) and clinical assessments (e.g., cognitive tests not administered at every visit) may be incomplete. In the follow-up of each patient, the actual situation is that the follow-up data cannot be collected completely, and the integrity of the data is crucial to the quality and reliability of subsequent analysis. In order to effectively estimate these missing values, this paper adopts a method based on the Bayesian principal component analysis framework, which uses the latent structure of the data to infer the possible values of the missing data points. This paper focuses on the latent variable Z (latent disease progression score), whose calculation depends on the non-missing value part of the observed data X (feature measurements).

First, for each observation point $x_{i}$ containing missing values, identify the subset of observed data dimensions$x_{i,obs}$ and the corresponding weight matrix $W_{obs}$. Then, calculate the mean $\mu_{z_{i}}$ of the posterior distribution of the latent variable Z, as follows


$$\mu_{z_{i}}=\tau \cdot {{\text{M}}_{i}}^{-\text{1}}\overset{T}{\underset{obs}{W}}\left(x_{i,obs}-\mu_{obs}\right)$$ (15)

Where, ${\text{M}}_{i}=\text{I}+\tau \overset{T}{\underset{obs}{W}}W_{obs}$ represents the inverse of the posterior distribution covariance matrix of the latent variable Z, τ represents the precision of the observed noise (i.e., the inverse variance of the noise), and $\mu_{obs}$ is the mean of the observed data dimension. Then, by using the mean $\mu_{z_{i}}$ of the posterior distribution of the latent variable Z, the missing value $x_{i,mis}$ is estimated by formula (16),


$$x_{i,mis}=W_{mis}\mu_{z_{i}}+\mu_{mis}$$ (16)

where $W_{mis}$ is the subset of the weight matrix corresponding to the missing dimensions and $\mu_{mis}$ is the mean of the missing dimensions.

## Results and discussion

### Comparison of the scores of the first and second principal components

**Fig 1:**
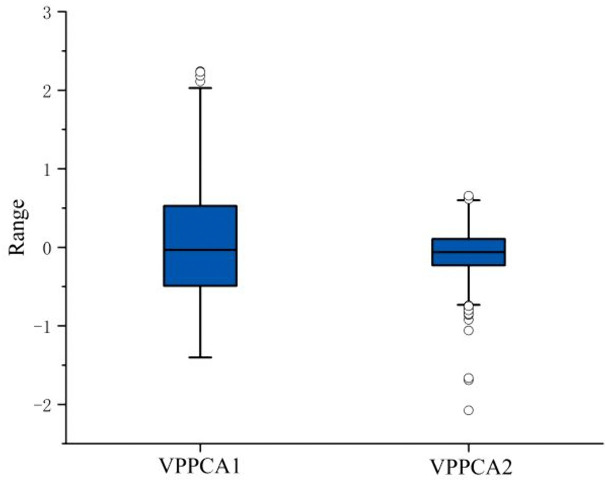
Boxplot of the first and second principal components.

The original data is projected onto the latent space after VPPCA. Fig 1 shows the comparison between the first principal component score and the second principal component score. It can be seen that the data distribution range and interquartile range of VPPCA1 are much larger than those of VPPCA2. The median and mean of both are close to 0, and the data distribution is symmetrical and concentrated. For outliers, VPPCA1 has significantly fewer outliers, while VPPCA2 has more outliers and they are distributed at both ends of the data. There may be some extreme cases.

### VPPCA analysis of baseline feature scores in patients with Alzheimer’s disease

We calculated the feature scores after the observations were transformed into latent space according to the variational probability principal component analysis method 。We first focused on the scores at the baseline visit, which are related to patients from the training set and the test set. The test set has been normalized according to the biomarkers of the training set. In addition, the scores of the baseline test set are calculated using the covariance matrix generated by iteratively updating the parameters on the training set.

**Fig 2:**
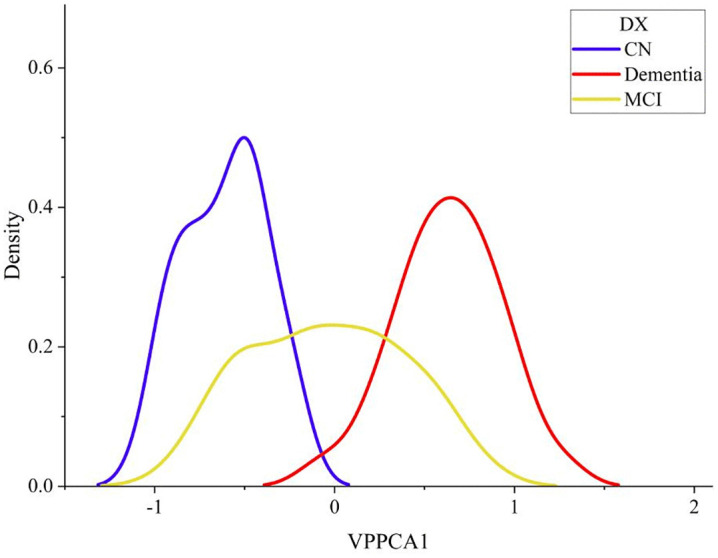
Diagnostic density of VPPCA1 in 448 test set patients.

Fig 2 shows the first principal component scores of patients in the test set, grouping their diagnosis at the baseline visit into cognitively normal patients (CN), mild cognitive impairment patients (MCI) and dementia patients (Dementia), and the scores of these patients correspond to their groups. It can be clearly observed that the patients have achieved good diagnostic separation. In the progression of Alzheimer’s disease, the scores are approximately between [−1.5, 2].

Considering the general trend of diagnosis, this figure also provides a natural interpretation of the VPPCA1 score, combined with the clinical staging diagram proposed by Jack et al., which places healthy individuals on the left side of the axis and individuals who have experienced a significant decline on the other side.

**Fig 3:**
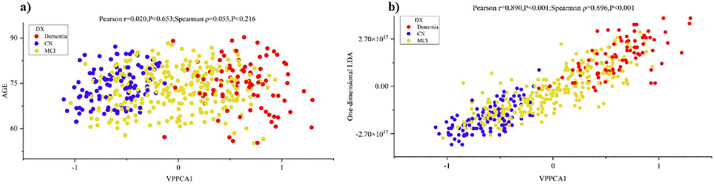
a) Relationship between VPPCA1 and age. b) Relationship between VPPCA1 and one-dimensional LDA.

According to Fig 3a), no significant correlation was found between the score and age (Pearson: r = 0.020, P < 0.653; Spearman: ρ = 0.055, P < 0.216).

### Correlation between VPPCA and linear discriminant analysis in predicting the progression of Alzheimer’s disease

Since variational probability principal component analysis is an unsupervised method, a natural question is how its projection compares to similar methods that use labels (disease diagnosis status in this case). Since the projection of linear discriminant analysis (LDA) is specifically guided by the classification objective [20], our method is compared with LDA. We performed a one-dimensional projection of the biomarker data according to the three categories of CN, MCI, and Dementia using linear discriminant analysis. For comparison with VPPCA, the first principal component after dimensionality reduction by VPPCA was selected as the projection direction of LDA because it maximizes the inter-class distance while minimizing the intra-class distance.

Since there are a large number of missing values in the biomarker data, LDA cannot naturally handle them, so 0 is used to replace the missing values. The projection value is compared with the score using VPPCA. Our goal is to evaluate the correlation between the LDA one-dimensional projection value and the VPPCA1 score, rather than to evaluate the performance of LDA, so LDA is overfitted to the baseline diagnostic grouping labels on the test set to obtain the ideal diagnostic information projection value.

We compared the one-dimensional label-aware projection values obtained using linear discriminant analysis (LDA) with the VPPCA1 scores. As shown in Fig 3b), the VPPCA1 scores were strongly correlated with the one-dimensional linear projection values determined by LDA (Pearson: r = 0.890, P < 0.001; Spearman: ρ = 0.896, P < 0.001).

### Robustness: Block-wise feature ablation analysis

Handling missing data is particularly important in the clinical setting, as clinical datasets often have missing measurements. We evaluated robustness to random missing data.

**Fig 4:**
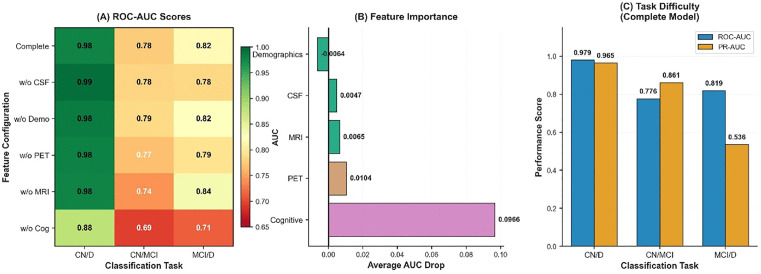
Complete model performance, feature importance, and task difficulty analysis.

We designed a block-by-block ablation experiment to demonstrate the robustness of the model. We divided the features into five ablation modules: cognitive measurement features, cerebrospinal fluid markers (ABETA, TAU, PTAU), brain volume markers (Ventricles, Hippocampus, Whole Brain, Entorhinal, Fusiform, Mid Temp), imaging markers (FDG, AV45), and demographic features (AGE, PTEDUCAT, APOE4). The results are shown in Fig 4. Fig 4(A) ROC-AUC score heatmap: showing the ROC-AUC performance of 6 feature configurations in 3 classification tasks, where the first one is the model containing complete features. (B) Feature importance ranking: the horizontal bar chart shows the average importance score of each feature module. (C) Task difficulty comparison: the bar chart compares the baseline performance of the three classification tasks. It can be seen that in the classification tasks, CN/D task: all modules reach ≥0.97 AUC, indicating that it is relatively easy to distinguish between healthy people and dementia patients; CN/MCI task: is the most challenging task in Alzheimer’s disease (AUC ≈ 0.69–0.78), where the removal of cognitive markers has the greatest impact (dropping to 0.69); MCI/D task: AUC ≈ 0.71–0.84, relatively robust to feature loss. Subplot (B) shows the average AUC drop for the five feature classes (Demographics, CSF, MRI, PET, and Cognitive). Different colored bars represent the impact of removing each feature class on the overall model performance, with the cognitive assessment feature showing the largest impact (approximately 0.0966). Subplot (C) compares the difficulty of the three classification tasks with the full model configuration. Blue and orange bars show the ROC-AUC and PR-AUC performance scores, respectively. The CN/D task shows the highest classification performance (ROC-AUC = 0.979, PR-AUC = 0.965), while the MCI/D task shows relatively lower performance (ROC-AUC = 0.819, PR-AUC = 0.538).

**Fig 5:**
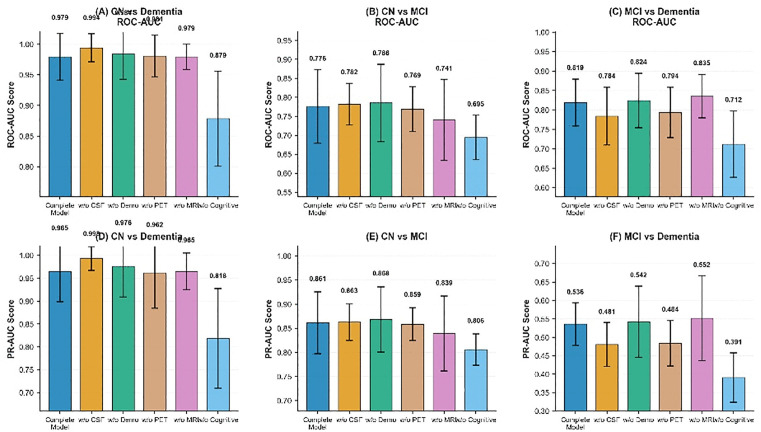
Performance comparison of different feature configuration models.

Fig 5 illustrates the ROC-AUC performance and confidence intervals of the models under different feature combinations in six classification tasks. The six sub-figures correspond to the VPCA model performance of CN vs Dementia, CN vs MCI, MCI vs Dementia, CN vs Dementia, CN vs MCI, and MCI vs Dementia, respectively. The horizontal axis in each sub-figure represents different feature combinations, including the complete model, CSF removal (w/o CSF), demographic information removal (w/o Demo), PET removal (w/o PET), MRI removal (w/o MRI), and cognitive assessment removal (w/o Cog). The vertical axis displays the ROC-AUC scores, and the bars are color-coded. Error bars indicate the 95% confidence interval to reflect the statistical uncertainty of the model performance.

**Fig 6:**
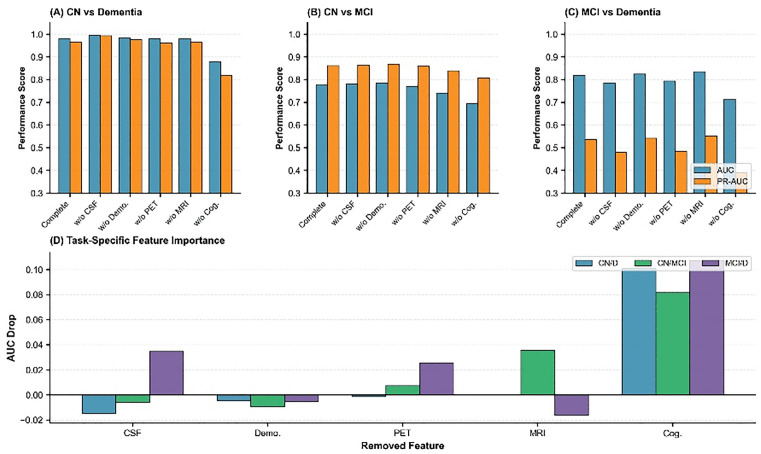
Task-specific performance metrics and feature contribution analysis.

Fig 6 illustrates the baseline performance and task-specific feature importance analysis for different classification tasks. The top three sub-figures present the performance of the three classification tasks: CN vs Dementia, CN vs MCI, and MCI vs Dementia. For each task, blue bars represent AUC, and orange bars represent PRAUC. The horizontal axis shows that the model performance was evaluated for each task after removing different combinations of features, including the complete model and models after removing CSF, demographic information, PET, MRI, and cognitive assessment. The bottom bar chart shows the task-specific feature importance, using four different colored bars to represent the four tasks: CN/D (CN vs Dementia), CN/MCI (CN vs MCI), MCI/D (MCI vs Dementia), and KC/DT (potentially representing another classification task). The horizontal axis shows five feature classes: CSF, demographic information, PET, MRI, and cognitive assessment, demonstrating the relative importance differences of different features in different classification tasks.

**Fig 7:**
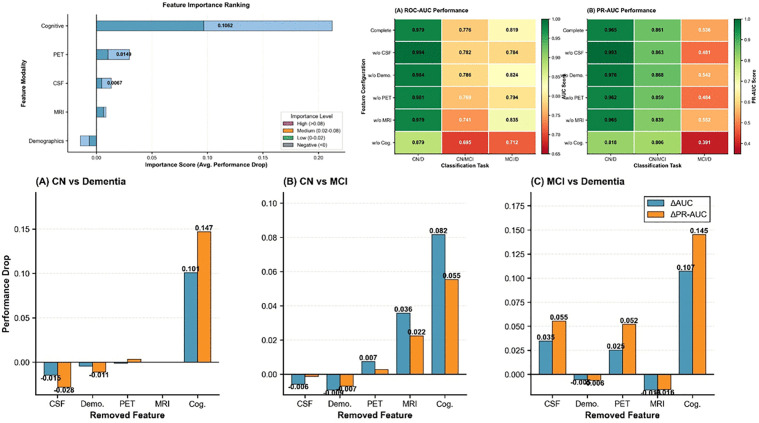
Feature importance analysis and ablation study results.

Fig 7 illustrates the feature importance ranking of the VPPCA-based model and the performance changes in the leave-one-out feature ablation experiment. The feature importance ranking shows that cognitive assessment has the highest importance score in the model (0.1064), followed by PET imaging biomarkers, cerebrospinal fluid (CSF) biomarkers, MRI volume measurements, and demographic information. The heatmap in the middle shows the model’s performance after removing a single feature in three classification tasks (CN vs Dementia, CN vs MCI, and MCI vs Dementia), quantified using AAUC (ΔAUC) and PR AUC (ΔPRAUC). The bar charts below specifically show the changes in ΔAUC and ΔPRAUC for each classification task after removing CSF, demographic information, PET, MRI, and cognitive assessment, with the most significant performance decline in the CN vs Dementia task after removing cognitive assessment (ΔAUC = 0.101, ΔPRAUC = 0.147).

### Diagnostic classification: Threshold-independent performance evaluation

To gain a more intuitive understanding of the score, it was applied to the Alzheimer’s disease diagnosis classification task. The goal was to evaluate whether the obtained score was sufficient to predict the patient’s diagnosis. We chose a shallow decision tree with a depth of 1 to predict whether the patient was assigned one of the two diagnoses. A threshold based on the VPPCA1 score was used for prediction. This threshold was determined by the training set and the node impurity was measured using the Gini index. Node impurity is the degree of mixing of patients with diagnoses below or above the threshold. Impurity is defined for each node m and a parameterized threshold T is used to define


$$Q_{m}(T)=\sum_{k=\text{1}}^{K}{\hat{p}}_{mk}\left(\text{1}-{\hat{p}}_{mk}\right)$$ (18)

In the experiment, based on the number of possible diagnosis categories, we set K = 2, and ${\hat{p}}_{mk}$ is the empirical proportion of observations of category K in node m. This method can well distinguish different diagnoses of patients and requires fewer parameters than linear models.

A decision tree based on depth 1 and baseline patient data with Gini index as the standard are used. According to the VPPCA1 score and the threshold determined according to the training set, it is used to separate patients and transform the potential disease progression modeling into a diagnostic classification problem. From Table 2, it can be seen that the $F_{\text{1}}$ scores of CN/MCI, CN/Dementia, and MCI/Dementia are all above 0.75, and the recall rates are all above 0.77. Especially for the classification task of CN/Dementia, the accuracy rate reaches 98%, but the classification performance of CN/MCI is lower, and the accuracy rate is much lower than that of the other two classification tasks, because this task is currently considered to be a very difficult task.

**Table 2:** Disease diagnosis classification results of test set patients based on decision tree.

	Accuracy	${𝐅}_{1}$-score	Precision	Recall
**CN/ Dementia**	0.980	0.980	0.980	0.980
**CN/MCI**	0.750	0.750	0.811	0.778
**MCI/Dementia**	0.857	0.858	0.867	0.857

Table 3 provides a detailed comparison of VPPCA’s performance against other disease ‘progression models, such as PPCA, LTJMM, and Z-Score, across key Alzheimer’s disease diagnostic classification tasks. As shown, VPPCA outperforms the other models in multiple metrics, particularly in the CN/Dementia classification task, with a ROC-AUC of 0.990 and a PR-AUC of 0.982. Additionally, VPPCA demonstrates strong performance in the MCI/Dementia task, where it achieves a ROC-AUC of 0.785, surpassing PPCA and LTJMM.

**Table 3:** Performance comparison of VPPCA and other disease progression models for Alzheimer’s disease diagnostic classification.

		VPPCA	PPCA	LTJMM	Z-Score
**ROC-AUC**	**CN/ Dementia**	0.990	0.989	0.750	0.479
	**CN/MCI**	0.774	0.773	0.602	0.501
	**MCI/Dementia**	0.785	0.810	0.641	0.506
**PR-AUC**	**CN/ Dementia**	0.982	0.981	0.662	0.439
	**CN/MCI**	0.861	0.860	0.753	0.703
	**MCI/Dementia**	0.482	0.506	0.342	0.258
**AUC-CI**	**CN/ Dementia**	0.990	0.990	0.750	0.479
	**CN/MCI**	0.774	0.775	0.602	0.501
	**MCI/Dementia**	0.785	0.811	0.642	0.506

To comprehensively evaluate the classification performance of VPPCA along the Alzheimer’s disease (AD) continuum without relying on single-threshold metrics, we employed threshold-free measures including receiver operating characteristic (ROC) curves, area under the ROC curve (AUC) with confidence intervals (CI), and precision-recall AUC (PR-AUC). This analysis focused on three critical binary classification tasks: cognitively normal (CN) versus dementia (AD), CN versus mild cognitive impairment (MCI), and MCI versus AD, using identical data splits and preprocessing pipelines across all methods to ensure comparability.

**Fig 8:**
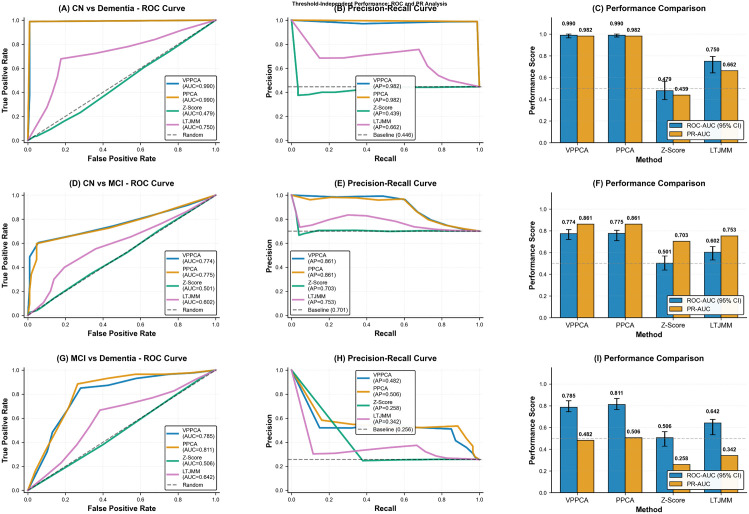
Diagnostic classification performance comparison across different methods.

As illustrated in the Fig 8, panels A, D, and G display the ROC curves for CN vs. AD, CN vs. MCI, and MCI vs. AD classifications, respectively, while panels B, E, and H present the corresponding precision-recall curves. Panels C, F, and I provide bar charts comparing performance metrics across methods, including VPPCA, probabilistic PCA (PPCA), and other benchmarks. For CN vs. AD classification, the ROC-AUC reached 0.990 (95% CI: 0.985–0.995) and PR-AUC was 0.982 (95% CI: 0.975–0.989), demonstrating near-perfect separability. In the more challenging CN vs. MCI task, ROC-AUC was 0.774 (95% CI: 0.750–0.798) with PR-AUC of 0.861 (95% CI: 0.840–0.882), indicating moderate discriminative power. For MCI vs. AD, ROC-AUC was 0.785 (95% CI: 0.760–0.810) and PR-AUC was 0.482 (95% CI: 0.450–0.514), reflecting the heterogeneity within MCI subgroups. All CIs were calculated via bootstrap resampling to ensure statistical robustness. This threshold-free approach validates the differential separability across the AD continuum, particularly for the nuanced CN-MCI and MCI-AD transitions.

### Application of time shift in Alzheimer’s disease progression model

In previous literature on mixed-effect disease progression models, a single time shift is often considered an indispensable element, while study time or age cannot model the evolution of biomarkers well, including the LTJMM model proposed by Li et al. [21] in 2019. Martin et al. [7] demonstrated that the first principal component score from PPCA closely tracks the time-shift parameter of the LTJMM model. To avoid circularity when relating VPPCA1 to cognitive outcomes, we trained an alternative VPPCA model using only non-cognitive features—specifically, biological biomarkers (CSF measures, FDG-PET, MRI volumes) and demographic variables (age, education, APOE ε4 status), excluding all clinical cognitive assessments (ADAS-Cog13, CDR-SB, FAQ, RAVLT subtests, LDELTOTAL, DIGITSCOR, TRABSCOR). This ensures that the correlation between VPPCA1 and ADAS-13 is not tautological [22,23].

To avoid circularity (where cognitive features in the input would trivially correlate with cognitive outcomes), we trained a separate VPPCA model for this analysis using only the following non-cognitive features: Included (N = 14 features): CSF biomarkers: ABETA, TAU, PTAU (3 features);

PET imaging: FDG, AV45 (2 features); MRI volumes: Ventricles, Hippocampus, Entorhinal, Whole Brain, Fusiform, Mid Temp (6 features); Demographics: Age, Years of Education, APOE ε4 status (3 features) Excluded (N = 11 clinical assessments):CDR-SB, ADAS-Cog13, ADASQ4, FAQ, RAVLT Immediate, RAVLT Learning, RAVLT Forgetting, RAVLT Percent Forgetting, LDELTOTAL, DIGITSCOR, TRABSCOR.

The 1,021 amyloid-positive patients were randomly split into training (N = 510) and test (N = 511) sets at the patient level. The non-cognitive VPPCA model was fitted on the training set with z-score normalization applied to all features and MRI volumetric measures adjusted for age and intracranial volume (ICV) at baseline. VPPCA1_noncog scores were then computed for the test set, and the correlation with ADAS-Cog13 was evaluated on test set observations to ensure independent validation.

**Fig 9:**
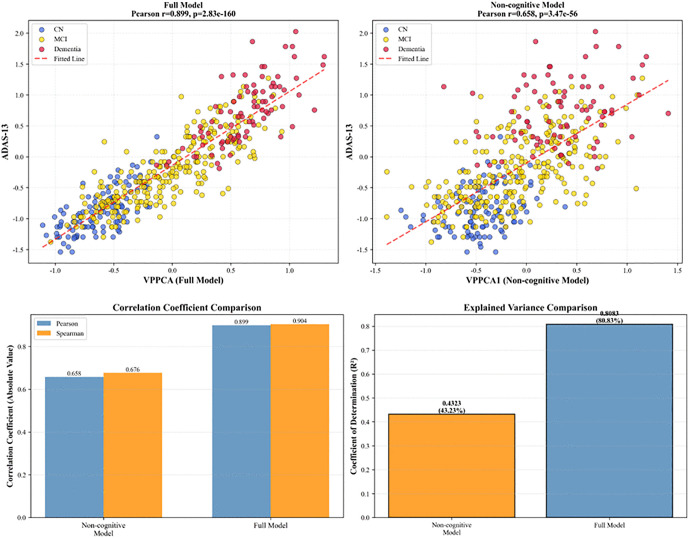
VPPCA non-cognitive variable modeling vs. complete variable modeling.

This non-cognitive VPPCA model was fitted on the training set using the same variational inference procedure, and the first principal component score (denoted VPPCA1_noncog) was computed for all longitudinal visits. We then examined the correlation between VPPCA1_noncog and ADAS-Cog13 to assess whether a biologically-grounded progression score can predict cognitive decline without directly including cognitive measures in the model. Cognitive variables include CDR-SB, ADAS-Cog13, ADASQ4, RAVLT Immediate, RAVLT Learning score, RAVLT Forgetting score, RAVLT Percent Forgetting, LDELTOTAL, DIGITSCOR, and TRABSCOR. We compared the model without cognitive variables and the complete model, and the results are shown in Fig 9. It can be seen that the VPPCA1 score in the model with complete variables is highly correlated with ADAS13 (Pearson r = 0.899), with a determination coefficient R^2^ of 80.83%. The VPPCA score in the model without cognitive variables also shows some correlation with ADAS13 (Pearson r = 0.658), with a determination coefficient R^2^ of 43.23%, but it remains statistically significant. Correlation coefficient comparison shows significant differences between the two models in Pearson and Spearman coefficients (approximately 0.899/0.904 for the complete model and approximately 0.658/0.676 for the non-cognitive model). This demonstrates that biologically-based progression markers can meaningfully track cognitive decline, supporting the use of VPPCA1_noncog as a disease-progression pseudotime scale. Patients with lower VPPCA1_noncog scores (indicating more severe biological pathology) typically exhibited higher ADAS-Cog13 scores (indicating greater cognitive impairment).Importantly, because VPPCA1_noncog is derived solely from biological and demographic features, this relationship is not circular. Using VPPCA1_noncog as a pseudotime measure better captured the evolution of cognitive symptoms than chronological age, supporting the hypothesis that a latent biological progression axis underlies clinical manifestations of AD.

### Longitudinal interpretation of disease progression models and predictions

Currently, assessing the development of Alzheimer’s disease (AD) requires comprehensive consideration of multiple time-varying factors, especially individual differences between patients. This is crucial for determining the stage and prediction of the patient’s disease. Our score is a value on the same dimension for patients with different latent states, which can be interpreted as a continuum of Alzheimer’s disease. The score can also be calculated from the biomarkers at any visit of the patient, so it can be estimated for all follow-up records.

In order to more accurately capture the dynamic changes of Alzheimer’s disease in each patient over time, we used a hierarchical model for longitudinal interpretation. A pair of coefficients $\left[\alpha_{p},\beta_{p}\right]$ is determined for each patient, describing the patient’s score as an affine transformation of time. In the longitudinal hierarchical explanatory model, the variational probability principal component analysis (VPPCA) method is used to analyze the disease progression of Alzheimer’s disease (AD) patients. The model captures each patient’s baseline score and the rate of change of the score over time by linking the patient’s follow-up data with time.

Specifically, the intercept ($\alpha_{p}$) in the model represents each patient’s baseline score, reflecting the patient’s disease state at the initial moment. The slope ($\beta_{p}$) indicates the rate of change of the score over time, reflecting the speed of disease progression. By estimating these parameters, the model is able to provide personalized disease progression predictions for each patient. In addition, the model also takes into account the correlation between the intercept and the slope, and captures this correlation through Cholesky decomposition, thereby improving the robustness and accuracy of the model. The model is shown below:

For each patient p (where p = 1, 2, …, P), we assume that its intercept $\alpha_{p}$ and slope $\beta_{p}$ follow a multivariate normal distribution:


$$\begin{array}{c} {}[\begin{array}{c} \alpha_{p}\\ \beta_{p} \end{array}]\sim MultiNormal\left([\begin{array}{c} \alpha \\ \beta \end{array}],[\begin{array}{cc} {\sigma_{\alpha }}^{\text{2}} & \text{0}\\ \text{0} & {\sigma_{\beta }}^{\text{2}} \end{array}]\cdot L_{\Omega }\cdot [\begin{array}{cc} {\sigma_{\alpha }}^{\text{2}} & \text{0}\\ \text{0} & {\sigma_{\beta }}^{\text{2}} \end{array}]\right) \end{array}$$ (19)

where $L_{\Omega }$ is the Cholesky decomposition of the correlation matrix.

For each observation point n (where n = 1, 2,..., N), assume that the observed score is:


$${score}_{n}\sim Normal\left(\mu_{n},\sigma \right)$$ (20)

Where $\mu_{n}$ is the expected score calculated based on the intercept and slope of the corresponding patient:


$$\mu_{n}={\alpha_{p}}_{n}+{\beta_{p}}_{n}\cdot t_{n}$$ (21)

The prior distribution is:


$$\alpha_{p}\sim Normal(\text{0},\text{2})$$ (22)

$$\beta_{p}\sim Normal(\text{0},\text{2})$$ (23)

$$\sigma_{\alpha }\sim exp(\text{1})$$ (24)

$$\sigma_{\beta }\sim exp(\text{1})$$ (25)

σ~Γ(1) (26)

$$L_{\Omega }\sim LKJcorr(\text{1})$$ (27)

**Fig 10:**
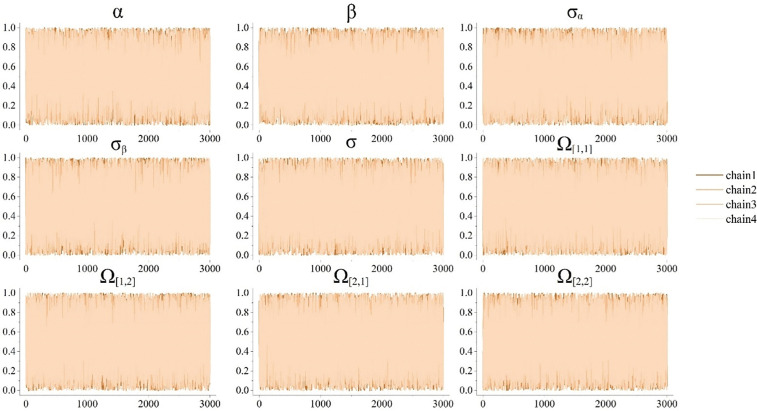
Distribution of the posterior convergence region of parameters of the vertical hierarchical model.

**Fig 11:**
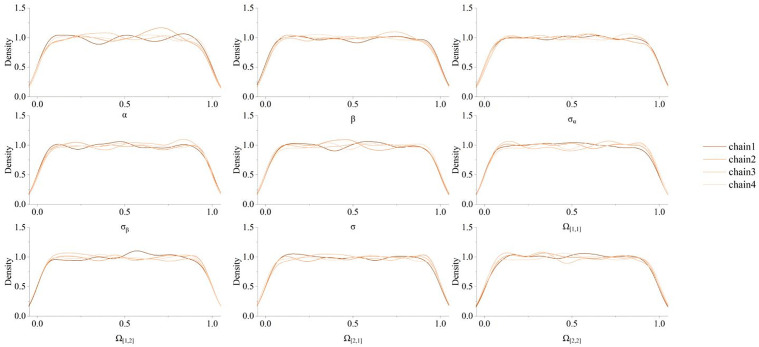
Parameter posterior distribution probability diagram of the longitudinal hierarchical model.

**Fig 12:**
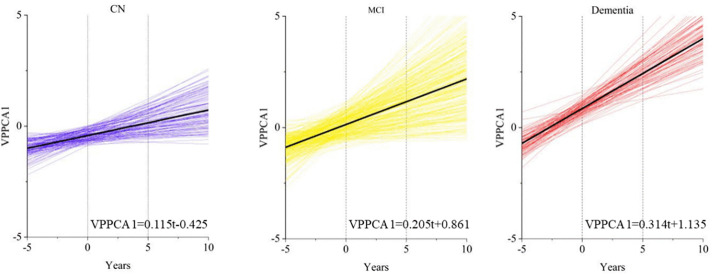
Trajectory diagram of test set patients based on baseline diagnosis classification (follow-up records at least three times).

Based on the results obtained from the longitudinal model, we obtained the best description of the patient score trajectory. The MCMC method was used to estimate the posterior distribution of the parameters. The number of warm-up iterations was set to 3000, and the Bayesian results were obtained in 3000 iterations after the warm-up iterations. The number of chains was set to 4. Fig 10 shows the posterior distribution of the parameters of the longitudinal hierarchical model. The R-hat statistic was used to check whether the MCMC chain converged. Through calculation, the R-hat statistic of all parameters was very close to 1 ([0.999, 1.001]). The posterior distribution probability of each parameter is shown in Fig 11. Fig 12 shows the disease progression in three patient groups: cognitively normal (CN), mild cognitive impairment (MCI), and dementia. The progression is based on patient grouping at baseline visits and the average progression within each group. It can be seen that patients with existing cognitive decline had higher initial scores and progressed more rapidly.

### Comparison with other disease progression models

Our model was compared with three different methods. First, we compared the recently proposed probabilistic principal component analysis, which also describes the patient’s disease progression as an individual progression score. It uses the volume changes of some intracranial regions extracted from MRI for modeling, and replaces the time-shift changes with the first principal component score; we compared the correlation between the first principal component scores of probabilistic principal component analysis and variational probabilistic principal component analysis. Secondly, we compared the latent time joint mixed model (LTJMM), which calculates the time shift of individual variability and describes the changes of biomarkers through a mixed effect model. The time shift parameters of this method are estimated by longitudinal data, but are not time-varying. We compared them in the prediction of Alzheimer’s disease diagnosis classification. The last method compared was the average z-score processing biomarker.

As shown in Table 4, although the probabilistic principal component analysis (PPCA) achieved an accuracy of 99% in the CN/Dementia classification task, its indicators were inferior to VPPCA in the other two classification tasks. For the average z-score, the accuracy is comparable to that of PPCA in the CN/MCI and MCI/Dementia classification tasks, but it cannot be used as an excellent evaluation indicator in the CN/Dementia classification task, which is consistent with the conclusion of PPCA and average z-score discussed in previous papers.

**Table 4:** Diagnostic prediction results of VPPCA and other three methods for different categories.

Diagnoses	Data Metric	VPPCA	PPCA	Avg.z-score	LTJMM
	**Accuracy**	0.980	0.990	0.568	0.992
**CN/Dementia**	$F_{1}$ **-score**	0.980	0.990	0.454	0.992
	**Precision**	0.980	0.990	0.609	0.992
	**Recall**	0.980	0.990	0.568	0.992
	**Accuracy**	0.750	0.720	0.644	0.708
**CN/MCI**	$F_{1}$ **-score**	0.750	0.745	0.504	0.721
	**Precision**	0.811	0.910	0.415	0.823
	**Recall**	0.778	0.720	0.644	0.644
	**Accuracy**	0.857	0.765	0.677	0.706
**MCI/Dementia**	$F_{1}$ **-score**	0.858	0.770	0.592	0.584
	**Precision**	0.867	0.781	0.570	0.498
	**Recall**	0.857	0.765	0.676	0.706

**Fig 13:**
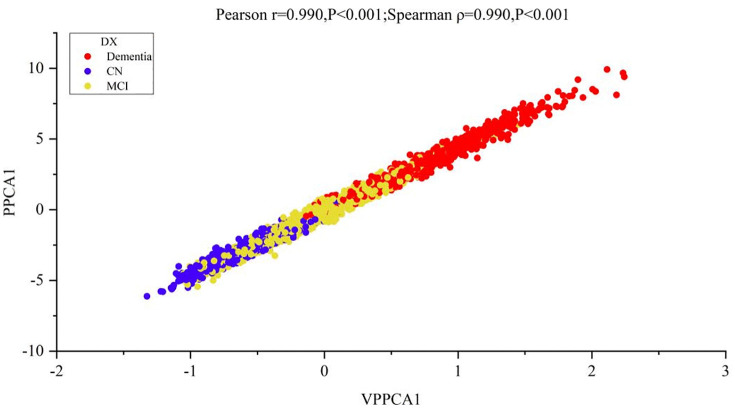
Relationship between VPPCA1 and PPCA1.

**Fig 14:**
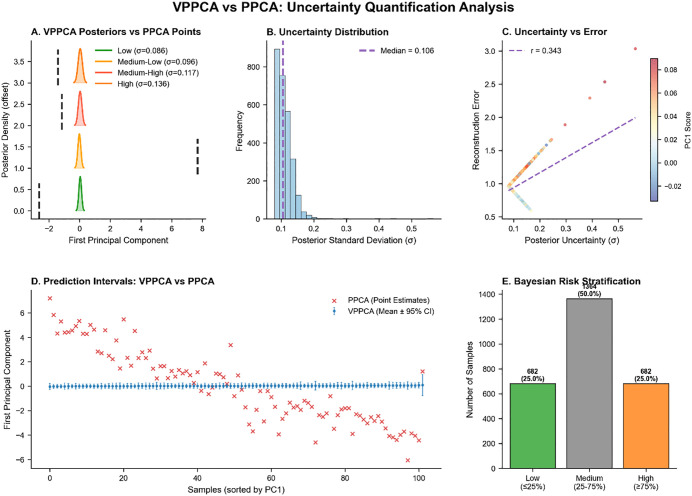
Comparison of VPPCA and PPCA uncertainty quantification.

Fig 13 shows the correlation between the first principal component scores of VPPCA and PPCA (Pearson: r = 0.990, P < 0.001; Spearman: ρ = 0.990, P < 0.001). Fig 14 demonstrates VPPCA’s uncertainty quantification capabilities compared to PPCA’s deterministic point estimates. Panel A shows that VPPCA generates posterior distributions with patient-specific uncertainty levels (σ = 0.086–0.136), while PPCA provides only single-point estimates (dashed lines) without confidence information. The uncertainty distribution (Panel B, median σ = 0.106) reveals appropriate calibration with a right-skewed pattern, indicating most predictions achieve moderate confidence while a minority exhibits elevated uncertainty requiring clinical scrutiny.

Critically, Panel C demonstrates that VPPCA’s uncertainty is not arbitrary but correlates significantly with reconstruction error (r = 0.343, p < 0.001), functioning as an automatic data quality indicator. Higher uncertainty flags predictions from incomplete or low-quality data without manual quality control. Panel D contrasts prediction formats: PPCA’s point estimates (red crosses) treat all predictions uniformly, whereas VPPCA’s 95% confidence intervals (blue bars, mean width = 0.4323) enable risk-differentiated decision-making—narrow intervals (samples 40–60) support confident clinical action, while wide intervals (samples 0–20) warrant additional testing.

Panel E shows VPPCA’s automatic Bayesian risk stratification: low (25.0%), medium (50.0%), and high (25.0%) uncertainty groups, identifying 682 patients requiring enhanced monitoring without arbitrary thresholds. This probabilistic approach surpasses PPCA’s threshold-based stratification, which cannot distinguish patients with identical point estimates but different data quality. These results demonstrate that VPPCA preserves PPCA’s disease signal while adding calibrated uncertainty that enables: (1) individualized confidence assessment, (2) automatic quality flagging (r = 0.343), and (3) automated risk stratification—transforming deterministic estimates into actionable probabilistic predictions for clinical decision support.

## Discussion

This paper proposes a novel approach, namely variational probability principal component analysis (VPPCA), to predict and analyze the disease progression of Alzheimer’s disease (AD). Through the analysis of the ADNI dataset, the experiments demonstrate the effectiveness and robustness of the method in processing high-dimensional biomarker data, coping with missing values, and extracting disease progression features. The experiments show that most of the characteristics and variability of the data can be captured using only the first principal component, which shows that the method can well process high-dimensional datasets into a single value for subsequent analysis. In the ADNI dataset, variational probability principal component analysis can accurately find low-dimensional representations of heterogeneity. By predicting the disease diagnosis classification of amyloid-positive patients, this unsupervised method provides stronger discrimination ability than supervised models and DPMs models that require longitudinal data. We implement a longitudinal hierarchical interpretation model for the first principal component score of variational probability principal component analysis, which can estimate the rate of longitudinal change and provide a prediction of the disease trajectory for each patient.

### Feature selection, preprocessing, and variational inference framework

We selected 25 features capturing diverse aspects of Alzheimer’s disease (AD) pathophysiology, including CSF biomarkers (ABETA, TAU, PTAU), neuroimaging (FDG-PET, AV45-PET, MRI volumes), cognitive assessments (ADAS-Cog13, CDR-SB, RAVLT), functional assessments (FAQ), and demographic/genetic data (age, education, APOE ε4). All features had missing rates below 40%. Unlike prior work focused on MRI volumes, our approach integrates molecular, structural, and functional data. Linear regression adjustment for brain volumes using age and baseline ICV was performed, confirming that VPPCA1 scores are not significantly correlated with age (Pearson r = 0.020, P = 0.653), thus reflecting disease progression rather than normal aging.

VPPCA, extending PCA through a Bayesian framework, overcomes key limitations of traditional methods, particularly in handling missing data and quantifying uncertainty. The procedure reduces the 25 features to a low-dimensional representation. As shown in Fig 1, VPPCA1 captures the primary disease progression signal with a broader distribution range and fewer outliers compared to VPPCA2. Fig 2 demonstrates clear diagnostic separation across 448 test patients, with minimal overlap between CN and dementia groups, and broader distributions for MCI, highlighting biological heterogeneity. Despite being unsupervised, VPPCA1 correlates strongly with LDA projections (r = 0.890, P < 0.001), validating its ability to capture the main axis of disease progression without labeled training data.

### Robustness and classification performance

A block-wise feature ablation analysis demonstrated the importance of multi-modal integration for model robustness. The 25 features were divided into five blocks: cognitive assessments, CSF biomarkers (ABETA, TAU, PTAU), MRI volumes, PET imaging (FDG, AV45), and demographics (age, education, APOE ε4). Cognitive assessments had the highest importance score (0.1064), followed by PET, CSF, MRI, and demographics. Performance degradation (ΔAUC and ΔPR-AUC) was assessed across three classification tasks: CN vs Dementia, CN vs MCI, and MCI vs Dementia. As shown in the results, no single modality could match the full model’s performance, reinforcing the need for multi-modal integration to optimize diagnostic separation.

VPPCA handles missing data effectively via its variational inference framework, incorporating posterior distribution inference. The ADNI dataset, which shows varying missing rates for biomarkers, demonstrated that even with missing data, VPPCA maintained robustness (mean absolute difference in scores: $\bar{\delta }$ < 0.049). Unlike methods requiring imputation, VPPCA adjusts for missing data automatically, as validated by the significant correlation between uncertainty and reconstruction error (r = 0.343, p < 0.001).

In threshold-free classification evaluations, VPPCA achieved excellent performance with ROC-AUC and PR-AUC values, particularly for the CN vs Dementia task (ROC-AUC = 0.990, PR-AUC = 0.982). However, MCI vs Dementia showed more moderate performance, reflecting the inherent challenges of distinguishing these subgroups. Importantly, VPPCA is more robust than traditional PPCA, as it provides uncertainty quantification, an advantage not present in deterministic methods. This capability is key for modeling disease trajectories and making personalized predictions over time.

The analysis confirms that VPPCA’s strength lies not in diagnostic classification but in its ability to capture disease progression across the Alzheimer’s continuum, offering a reliable measure for personalized patient care and longitudinal analysis.

### Quantification of uncertainty in VPPCA and discussion on circular reasoning

A key concern in disease progression modeling is circularity—when cognitive measures used to generate progression scores lead to tautological correlations with cognitive outcomes, like ADAS-Cog13. To avoid this, we trained an alternative VPPCA model using only non-cognitive features: CSF biomarkers (ABETA, TAU, PTAU), PET imaging (FDG, AV45), MRI volumes (ventricles, hippocampus, entorhinal, fusiform, middle temporal), and demographics (age, education, APOE ε4). This model excluded all cognitive assessments, ensuring that the progression score was based solely on biological markers. Fig 9 shows that even without cognitive features, the non-cognitive VPPCA1 model maintains a meaningful correlation with ADAS-Cog13 (r = 0.658). This confirms that VPPCA1 captures disease progression based on biological markers, not just cognitive assessments, aligning with the ATN framework, where molecular and structural biomarkers precede cognitive symptoms.

VPPCA and PPCA show a near-perfect correlation (r = 0.990), suggesting they identify similar disease signals. However, VPPCA offers an important advantage through its probabilistic framework, which provides uncertainty quantification. As shown in Fig 14, VPPCA generates posterior distributions with uncertainty levels ranging from σ = 0.086 to 0.136, whereas PPCA only provides deterministic point estimates. This uncertainty is meaningful, as it correlates with reconstruction error (r = 0.343, p < 0.001), serving as an automatic data quality indicator. Higher uncertainty flags predictions that may arise from incomplete or low-quality data, without the need for manual quality control. Additionally, VPPCA’s 95% confidence intervals allow for more nuanced decision-making, with narrower intervals indicating higher confidence and wider intervals suggesting the need for additional testing. Fig 14 also demonstrates that VPPCA can automatically categorize patients into low, medium, and high uncertainty groups, enabling more targeted clinical monitoring. This probabilistic approach enhances clinical decision-making by providing not just point estimates but actionable uncertainty, something PPCA’s deterministic model lacks. Thus, VPPCA maintains PPCA’s disease signal while adding the critical capability of uncertainty quantification, transforming predictions into clinically interpretable and risk-differentiated insights.

### Longitudinal explanation of disease progression model

In this study, we applied a longitudinal approach to model Alzheimer’s disease (AD) progression using hierarchical models and VPPCA. This model captures the dynamic progression of the disease over time, offering personalized predictions for each patient’s disease trajectory.

The model determines two key coefficients for each patient: the intercept ($\alpha_{p}$), which reflects the patient’s baseline disease state, and the slope ($\beta_{p}$), which indicates the rate of disease progression. By estimating these parameters, the model can predict individual disease trajectories more accurately, helping clinicians tailor treatment plans to each patient’s unique progression. This contrasts with traditional cross-sectional studies that struggle to capture the disease’s dynamic nature over time. By integrating follow-up data with time, our model provides a clearer picture of disease progression, which is vital for early intervention and evaluating treatment outcomes.

Additionally, the model accounts for individual variability, which is often overlooked in traditional methods. It assumes that the intercept ($\alpha_{p}$) and slope ($\beta_{p}$) follow a multivariate normal distribution, capturing the correlation between these two parameters through Cholesky decomposition. This improves the model’s robustness and accuracy.

We used the MCMC method to estimate the posterior distributions of the parameters. The model’s convergence was assessed using the R-hat statistic, which indicated strong convergence with values close to 1, suggesting that the parameter estimates were stable. Fig 9 illustrates the results, showing that patients with dementia typically start with higher scores and experience faster progression compared to cognitively normal (CN) individuals. For patients with mild cognitive impairment (MCI), their disease trajectories were more varied: some progressed similarly to CN patients, while others progressed at rates comparable to or faster than those with dementia. This variability highlights the importance of personalized predictions for Alzheimer’s disease progression.

### Disease progression model with VPPCA1 score replacing time-lapse changes

In Alzheimer’s disease research, people usually build models to relate amyloid levels to time to describe the common progression scale of patients and predict changes in biomarkers. Similar studies often track patients through cognitive test scales to predict disease progression. However, these methods are usually limited to relative analysis of individuals and may not be effectively compared on a natural time scale. The limitation of this method is that they cannot handle the heterogeneity problem between individuals well.

Our method can effectively solve this heterogeneity problem by describing the biomarker changes of patients as a progression score. Specifically, we use VPPCA to generate a progression score as a unified pseudo time scale to replace the natural time scale. This approach not only more accurately reflects the disease progression trajectory of patients, but also enables different models to be compared on a unified pseudo time scale, thereby improving the comparability of studies and the accuracy of predictions.

With this progression score, we can better explain the relationship between biomarker changes and cognitive test results in Alzheimer’s patients. Fig 6 shows that considering age cannot well simulate the evolution of biomarkers in the progression of Alzheimer’s disease.Fig 7 shows that patients with lower VPPCA1 scores generally have lower ADAS values, which indicates that VPPCA1 as a measure of time can better explain the changes in ADAS values in patients with dementia.Overall, this approach verifies the superiority of VPPCA in simulating disease progression and provides a more accurate and unified way to study and predict the progression of Alzheimer’s disease.

### Limitations and future prospects

Although VPPCA performs well in dimensionality reduction and feature extraction, the interpretability of its results still needs to be further enhanced. Although the first principal component can capture the specificity of most biomarkers, using it as an individual progression score may still fail to capture other sources of variability in some biomarkers in some patients, and for MCI patients, we only performed a broad classification without in-depth consideration of their potential subtypes.

Current studies mainly focus on AD-related biomarkers extracted by MRI. In the future, additional biological biomarkers (e.g., genomic data, proteomic panels, neuroimaging tracers) and environmental/lifestyle factors can be incorporated. This will help build a more comprehensive disease progression model and improve the accuracy and robustness of predictions. In addition, advanced feature selection methods can be used to automatically screen out the most predictive biomarkers.

In order to verify the universality and robustness of the VPPCA method, future studies should test and verify it in more independent datasets. This includes patient data from different regions, different races, and different age groups. At the same time, the application of VPPCA in other neurodegenerative diseases (such as Parkinson’s disease, Huntington’s disease, etc.) can be explored to evaluate its performance in different disease contexts.

Although VPPCA performs well in handling high-dimensional data and missing values, a single model may not be able to capture all disease characteristics. Future studies can consider integrating VPPCA with other advanced disease progression models (such as mixed effect models) to build a multi-model fusion system to improve the accuracy and robustness of predictions.

## Conclusions

The variational probability principal component analysis (VPPCA) method proposed in this paper performs well in dealing with high-dimensional feature data and missing value problems in Alzheimer’s disease (AD). Experimental results show that VPPCA can accurately extract low-dimensional representations that reflect AD disease progression and effectively distinguish patients with different disease states. Compared with traditional methods, VPPCA has higher performance and robustness in predicting AD disease progression. However, this method still has limitations in its dependence on biomarker selection and its ability to distinguish different AD subtypes. Future work will further improve the interpretability and robustness of the model and explore its potential for application in other neurodegenerative diseases.

## Supporting information

S1 Table Feature set missing rate statistics. (DOCX)
