# Supplementary material for: Disease progression modeling of Alzheimer’s disease based on variational probability principal component analysis
Source: PLoS One. 2026 Mar 30;21(3):e0342549. doi: 10.1371/journal.pone.0342549 (PMC13035165; doi:10.1371/journal.pone.0342549)
Supplement: S1 Table — (DOCX) [file pone.0342549.s001.docx]

**Supporting information**

**S1 Table**

| **Features** | **Total sample size** | **Number of missing samples** | **Missing rate** |
| --- | --- | --- | --- |
| **ABETA**  **TAU**  **PTAU**  **FDG**  **AV45**  **Hippocampus**  **Ventricles**  **Whole Brain**  **Entorhinal**  **Fusiform**  **Mid Temp**  **ADAS13**  **CDRSB**  **ADASQ4**  **RAVLT. immediate**  **RAVLT. learning**  **RAVLT. forgetting**  **RAVLT. perc. forgetting**  **LDELTOTAL**  **DIGITSCOR**  **TRABSCOR**  **FAQ**  **AGE**  **PTEDUCAT**  **APOE4** | 1021  1021  1021  1021  1021  1021  1021  1021  1021  1021  1021  1021  1021  1021  1021  1021  1021  1021  1021  1021  1021  1021  1021  1021  1021 | 189  189  189  240  406  132  56  39  155  155  155  7  0  1  3  3  3  4  0  397  36  7  1  0  14 | 18.51%  18.51%  18.51%  23.50%  39.76%  12.93%  5.48%  3.82%  15.18%  15.18%  15.18%  0.68%  0.00%  0.10%  0.29%  0.29%  0.29%  0.39%  0.00%  38.89%  3.53%  0.68%  0.10%  0.00%  1.37% |
